# A Structure Fidelity Approach for Big Data Collection in Wireless Sensor Networks

**DOI:** 10.3390/s150100248

**Published:** 2014-12-25

**Authors:** Mou Wu, Liansheng Tan, Naixue Xiong

**Affiliations:** 1 Department of Computer Science, Central China Normal University, Wuhan 430079, China; E-Mails: mou.wu@163.com (M.W.); l.tan@mail.ccnu.edu.cn (L.T.); 2 School of Information Technology, Jiangxi University of Finance and Economics, Nanchang 330013, China; 3 School of Computer Science, Colorado Technical University, Colorado Spring, CO 80907, USA

**Keywords:** data collection, spatial correlation, wireless sensor network, structure fidelity

## Abstract

One of the most widespread and important applications in wireless sensor networks (WSNs) is the continuous data collection, such as monitoring the variety of ambient temperature and humidity. Due to the sensor nodes with a limited energy supply, the reduction of energy consumed in the continuous observation of physical phenomenon plays a significant role in extending the lifetime of WSNs. However, the high redundancy of sensing data leads to great waste of energy as a result of over-deployed sensor nodes. In this paper, we develop a structure fidelity data collection (SFDC) framework leveraging the spatial correlations between nodes to reduce the number of the active sensor nodes while maintaining the low structural distortion of the collected data. A structural distortion based on the image quality assessment approach is used to perform the nodes work/sleep scheduling, such that the number of the working nodes is reduced while the remainder of nodes can be put into the low-power sleep mode during the sampling period. The main contribution of SFDC is to provide a unique perspective on how to maintain the data fidelity in term of structural similarity in the continuous sensing applications for WSNs. The simulation results based on synthetic and real world datasets verify the effectiveness of SFDC framework both on energy saving and data fidelity.

## 1. Introduction

Wireless sensor network (WSN) has been well-suited for use with a variety of applications including environmental monitoring, biological detection, smart spaces, and battlefield surveillance. These applications can be divided into two broad categories in terms of the way of data collection based on the observer interest: event-based detection and continuous data sampling. In event-based detection, the sensors report the data expected to collect to the sink only when an event of interest occurs. It means that the significant characteristic of the event-based detection in WSNs is delay intolerant and error sensitive. In other words, the data delivered to the sink need to be reliable once an emergency occurred within the sensing range of sensors is detected in real time. In this context, the detection success rate of interested events is crucial to the efficiency of the application.

Coverage-based scheduling methods can achieve full coverage of the target area by finding the disjoint cover sets of sensor nodes, and thus greatly improve the event detection rate. The network lifetime can be increased due to only one cover set is active and send their readings, while the remainder of the nodes goes to sleep and waits for the next time to be roused. To avoid the area uncovered, it is necessary to guarantee that the number of sensor nodes in each set is enough to cover the whole network.

In the continuous data sampling applications, the local measures in the sensor node's sensing area are regularly sampled and reported, such as the ambient temperature and humidity. After receiving all samples, the sink generates a data snapshot of the area at one point in time. Some applications may be more tolerant to discrepancies in the sensed values, not demanding the receiving data without any error. In a general case, the observers are more focused on the evolving process or spatial structure evolution of certain physical phenomenon in the monitored area. Such frequent sampling behavior expends more energy than the event detection. Thus reducing energy waste is even more important to the continuous data sampling applications. Unlike the event-based detection, the continuous data sampling applications are delay and error tolerant. Differences between them lead to that the coverage-based scheduling methods are unusable for the applications characterized by continuous sampling.

WSNs have great potential that are exploited for many applications in real world scenarios. However, it has been concluded that the problems of energy constraint and data redundancy emerge inevitably in the course of designing an application of WSNs, especially for high-density large scale network.

First, due to the relative less energy consumption on computation and sensing, wireless communications resulting from data delivery consume significant amount of power of sensor nodes only equipped with limited battery. In order to achieve longer lifetime, one of design challenges for WSNs is to reduce the amount of communication as much as possible without sacrificing measurement integrity.

Second, to satisfy full network coverage requirements [1] and compensate for the impact of nodes failure [2,3], sensor nodes are deployed with much greater density than is needed. Therefore, for the general case in which sensor nodes is highly over-deployed, it is common that nodes redundancy results in data redundancy, whereas we can infer the degree of node redundancy through analyzing the sensed data set. Further, the redundant sensor nodes can be scheduled to the sleep mode to save energy when the predefined observation fidelity is satisfied. The problem behind this approach is how to determine the observation fidelity. Much of existing work in this area is still based on the error sensitivity approach, where the mean square error (MSE) is considered as the sole type of distortion. The inherent disadvantage of this method, however, is that MSE dose not provide a good approximation of perceived structural information in the continuous data sampling applications. One distinct example is presented in research for image quality assessment [4], where larger MSE dose not show lower structure level on the test images. As mentioned before, the over-deployed sensor nodes are high correlated or structured, and thus meaning that the sampling data collected by spatial correlated nodes have strong dependencies carrying important structure information. We attempt to exploit the dependencies to reduce the number of nodes required to work for sampling and data transmission. Such reduction is bound to save energy and prolong the network lifetime of WSNs.

In this paper, we propose a novel approach to implement sensor scheduling by maintaining the continuous sampling data fidelity that is defined by the structural similarity successful applied to image quality assessment methods. The goal of image quality assessment research is to provide a direct approach that quantifies the similarity or difference between the test and reference images. The simplest implementation to achieve the goal is the error sensitivity approach, which determines a better quality image based on lower MSE. Some limitations on the traditional approach have been highlighted by many previous researchers from image processing area [5–7], e.g., quality definition problem, decorrelation problem and cognitive interaction problem. These problems also exist in the quality assessment of the continuous sampling applications for WSNs. A new philosophy for the assessment of image quality is the Structural SIMilarity (SSIM) proposed in [4,8,9], which calculate the structural similarity index of the degraded images by incorporating known human visual system (HVS) properties. With the method, the structural information of image signals consisting of abundant pixels exhibiting high spatial correlation can be extracted. Despite a big MSE relative to the reference image, the tested image still obtains a very high fidelity value as long as the most of structural information of the reference image can be preserved. Using the structural similarity to process large scale data in WSNs, especially when data is continuous sampled, it is unquestionable that we can get benefits in energy saving. There are two strong motivations for this:

- The extracted structural information is just the main concern in the continuous data sampling applications for WSNs. Such as the application domain that need to capture the data structural changes is highly adapted with the SSIM approach, which is inapplicable in event detection because of the character of error sensitivity.
- The limitations from the error sensitivity approaches are avoided when the SSIM index is used to be a data fidelity assessment criterion. In this way, we refer the data collected by sensor nodes at any time as a tested image to judge the node's contribution to the whole data collection. So that, only a part of sensor nodes need to work and sample while the senor nodes with a negligible contribution will enter a sleep mode.

Inspired by the above ideas, we propose a structure fidelity data collection (SFDC) framework leveraging the spatial correlations between nodes to reduce the number of the active sensor nodes while maintaining the low structural distortion of the collected data. The ultimate goal of this paper is to save energy in the continuous data sampling applications for WSNs by designing a novel node scheduling method, which considers the data fidelity in term of spatial structure instead of the traditional MSE. To get the most out of spatial correlation, our framework is based on the spatial clustering algorithm and splitted into two phases: the learning phase and data collection phase. During the learning phase, all sensor nodes report their values to the sink. The sink assigns the sensor nodes with close distance and similar trend of the historical data to the same cluster. Within each cluster, the one with the least mean deviation on the readings is selected as the cluster head (CH). In order to apply SSIM index to better in sensor scheduling, we develop a model to calculate a node contribution to the cluster in term of data structural similarity. When given a predefined structure threshold, the CH can make the scheduling decisions on cluster members' work/sleep during the subsequent data collection phase. Therefore, our SFDC framework is well aware of the spatial correlation between nodes and can achieve significant energy saving without too much structure loss.

The rest of this paper is organized as follows. In Section 2, we discuss the related works on data collection techniques for various applications in WSNs. In Section 3, we introduce the image quality assessment approach based on the structural similarity. Section 4 presents our structure fidelity data collection framework, where a clustering algorithm maximizing the utility of the structure fidelity approach and the nodes scheduling scheme guaranteeing the level of structural fidelity are proposed. Moreover, in order to avoid the structure index instability resulting from the change of the inter-node relationships, we propose an adaptive data collection method to adjust the data collection period against the cluster structure deterioration. Simulation results are provided in Section 5 to verify the correctness of SFDC framework both on energy saving and data fidelity and show the effectiveness of adaptive data collection. Finally, Section 6 presents the conclusions of the whole paper and future works.

## 2. Related Work

Many data collection approaches aimed to reduce energy consumption have been proposed for wireless sensor networks recently. LEACH (Low-Energy Adaptive Clustering Hierarchy) [10] is the most famous clustering-based data collection protocol that utilizes randomized rotation of local cluster heads to evenly distribute the energy load among the sensors in the network. Since LEACH uses localized coordination to enable scalability and robustness for dynamic networks, and incorporates data fusion into the routing protocol, it is not surprising that the amount of information that must be transmitted to the base station would be greatly reduced. Thereafter, a large number of data collection approaches based on clustering emerged as required. A centralized version of LEACH is LEACH-C proposed in [11]. Unlike LEACH, where nodes self-configure themselves into clusters, LEACH-C utilizes the base station for cluster formation. By using a central control algorithm, the base station selects the nodes with residual energy above the average node energy throughout the network as the cluster head nodes. A modification to LEACH is presented in [12], in which a two-level hierarchy clustering protocol (TL-LEACH) is designed for sensor networks where end user wants to remotely monitor the environment. TL-LEACH permits to better distribute the energy load among the sensors in the network especially when the density of network is higher. The impact of heterogeneity of nodes in terms of their energy in WSNs that are hierarchically clustered is studied in [13]. Adapting this approach, an energy efficient heterogeneous clustered (EEHC) scheme for WSNs based on weighted election probabilities of each node to become a CH according to the residual energy in each node is proposed.

Clustering techniques have an added benefit that provide an architectural framework for exploring data correlation in sensor networks. Some recent research on spatial clustering investigates the correlation measurement on sensor data in a highly correlated region. By using the spatial distance of sensors as the judgment of correlation, an approach presented in [14] defines a weight for each sensor's data which depends on the distance from the sample position to the target position. The Clustered AGgregation (CAG) proposed in [15] provides a mechanism that reduces the number of transmissions by calculating the deviation between different sensor readings to measure the spatial correlation. The cluster construction behind this concept is that a node is included in the cluster only when the deviation between the readings of it and the cluster head is less than the a user-provided error threshold, otherwise this node becomes a new cluster head. In the response phase, CAG transmits a single value per cluster, where only the cluster heads contribute to the data aggregation. By focusing on a few representative values rather than a large number of redundant data, CAG achieves a significant energy saving at the expense of a negligible quality. Another judgment method proposed in [16] is that a probability distribution function and correlation coefficients of nodes readings are used as a means of showing the representation of spatial correlation in the sensor network data model. A common feature in the approaches described above is that the cluster head nodes are selected stochastically. This raises the obvious problem that the selected cluster head is not best one for performing the high quality data collection in a specified application. In our SFDC framwork, the cluster head is the one that achieve the maximization of structure fidelity in the cluster data model. Thus overcoming the shortcoming of the random selection of cluster heads. Both the spatial distance and the correlation coefficient are considered in the design of our cluster construction such that the clustering algorithm provides a better foundation for obtaining the high structure fidelity.

The cluster head selection approach based on the dominating set theory is proposed in [17], the authors of which define a weight to calculate the spatial correlation between sensor data. When the clusters are constructed, the data sampled in each cluster head have very high correlation with the data sampled in its cluster members. Consequently, only the cluster heads that can represent the data features of its members need to do the data sampling work, which means the data transmitted in the sensor network are reduced remarkably without any extra data aggregation algorithm. However, the biggest problem for large-scaled WSNs is a massive amount of valuable information from member nodes cannot be found by the end users. In [18], an adaptive sampling approach (ASAP) is developed for energy efficient periodic data collection in WSNs. In ASAP, the samplers is composed of a dynamically changing subset of the nodes in which the readings are directly collected, whereas the readings of non-samplers are predicted through the use of probabilistic models that are locally and periodically constructed. ASAP can be effectively used to increase the network lifetime while avoiding the data loss from non-sampler nodes such that keeping the high quality of the collected data.

Another approach of energy-efficient data collection in WSNs is data prediction technique leveraging the spatial or temporal correlation between sensor data. The data prediction applying in WSNs has a tradeoff between reducing communication cost and limiting prediction cost. Based the analysis for the performance of the tradeoff in [19,20], an energy-efficient framework for clustering-based data collection is proposed in [21], where the benefit of adaptive scheme to enable/disable prediction operations is exploited. To improve prediction accuracy, authors of [22,23] perform prediction of data based on the multivariate correlation and multiple linear regression methods, respectively.

Our purpose of organizing sensor nodes into clusters is to realize the energy-efficient wake-up/sleep scheduling scheme by which some active nodes to provide sensing services, while the others are inactive to conserve their energy For nodes scheduling problem, many attempts have mainly focused on getting complete coverage of the monitored area, where a target of interest is within the sensing range of at least one sensor. A survey on target coverage problem in WSNs is presented in [24,25]. In [26] authors propose a target coverage scheduling scheme based on a genetic algorithm that can find the optimal cover sets to extend the network lifetime. In each cover set, a sufficient number of sensor nodes cover the specified targets by using the evolutionary global search technique. The problem of proper scheduling and putting unnecessary sensor nodes into sleep mode was also explored in [27]. The proposed scheme is a kind of an adaptive/periodic on-off scheduling scheme in which sensor nodes use only local information to make scheduling decisions. A sensing topology management strategy proposed in [28] have similar characteristics with our approach in terms of exploiting redundancy for continuous data sampling applications and no a-priori statistical assumptions on the underlying phenomenon need to be made. By introducing the finite-dimensional Hilbert space framework of sensors as random variables, sensor locations map onto vectors in this Hilbert space, and inner products between them are defined by the correlation structure of the sensed physical process. As a consequence of adopting the methodology, the number of disjoint sets of sensors can be maximized (or equivalently, the average number of sensors in each set is minimized), while ensuring that each one can provide no more than the user specified distortion in the data sampling period. However, the proposed algorithms do not consider the cluster-based networking architecture that proved to have more advantages than other network model such as energy-saving, scalability, ease of data fusion and robustness. Moreover, the method of randomly selecting the candidate nodes into the active set is not optimal for achieving the minimum expected distortion. Instead, our active nodes selection algorithm maintains the biggest increase in the structure fidelity for each addition.

## 3. Structural Similarity to Image Quality Assessment

In this section, we introduce the image fidelity metric that quantifies the distortion of a test image by comparing it with a reference image assumed to have perfect quality. How to assess the quality of a distorted image is the key issue in image processing applications. A number of metrics from different aspects have been proposed, e.g., visible errors, human perception and the performance of image compression algorithm. Since the human visual system is highly sensitive to the structural information, a new philosophy to compare the structures of the reference and the distorted images is proposed in [4,8,9].

Let *x* = {*x_i_*∣*i* = 1, 2, ⋯ , *N*} and *y* = {*y_i_*∣*i* = 1, 2, ⋯, *N*} be the reference and distorted image signals, where *i* is the sample index and *N* is the number of signal samples. The structural similarity index between signals *x* and *y* can be defined by:

(1) SSIM(x,y)=l(x,y)c(x,y)s(x,y)

where the three functions *l*(*x, y*), *c*(*x, y*) and *s*(*x, y*) represent the luminance, contrast and structure comparison measures between *x* and *y*, respectively.

Let *μ_x_*, *μ_y_*, $\sigma_{x}^{2}$, $\sigma_{y}^{2}$ and *σ_xy_* be the mean of *x*, the mean of *y*, the variance of *x*, the variance of *y*, and the covariance of *x* and *y*, respectively. Here the mean and the variance of a signal are treated as estimates of the luminance and the contrast of the signal. The covariance can be considered as a measurement of how much one signal is changed nonlinearly to the other signal being compared. The luminance, contrast and structure comparison measures are then given as follows:

(2) $$l\left(x,y\right)=\frac{2\mu_{x}\mu_{y}}{\mu_{x}^{2}+\mu_{y}^{2}},\;c\left(x,y\right)=\frac{2\sigma_{x}\sigma_{y}}{\sigma_{x}^{2}+\sigma_{y}^{2}},\;s\left(x,y\right)=\frac{\sigma_{xy}}{\sigma_{x}\sigma_{y}}$$

where

(3) $$\mu_{x}=\bar{x}=\frac{1}{N}\overset{N}{\underset{i=1}{\text{∑}}}x_{i},\mu_{y}=\bar{y}=\frac{1}{N}\overset{N}{\underset{i=1}{\text{∑}}}y_{i}$$

(4) $$\sigma_{x}^{2}=\frac{1}{N-1}\overset{N}{\underset{i=1}{\text{∑}}}{\left(x_{i}-\bar{x}\right)}^{2},\sigma_{y}^{2}=\frac{1}{N-1}\overset{N}{\underset{i=1}{\text{∑}}}{\left(y_{i}-\bar{y}\right)}^{2}$$

(5) $$\sigma_{xy}=\frac{1}{N-1}\overset{N}{\underset{i=1}{\text{∑}}}\left(x_{i}-\bar{x}\right)\left(y_{i}-\bar{y}\right)$$

The Equations (1) and (2) are combined and the SSIM index can be rewritten by

(6) $$\text{SSIM}\left(x,y\right)=\frac{4\mu_{x}\mu_{y}\sigma_{xy}}{\left(\mu_{x}^{2}+\mu_{y}^{2}\right)\left(\sigma_{x}^{2}+\sigma_{y}^{2}\right)}$$

Based on the Equation (6), the SSIM index has the following three properties:

(1) Symmetry: *SSIM* (*x*, *y*) = *SSIM* (*y*, *x*).
(2) Boundedness: *SSIM* (*x*, *y*) ∈ [−1,1] since he three components of Equation (1) rang from [0,1], [0,1] and [−1,1], respectively.
(3) Unique maximum: *SSIM* (*x*, *y*) = 1 if and only if *x* = *y* (in discrete representations, *x_i_* = *y_i_* for all *i* = 1, ⋯, *N*.

In practice, we usually want to evaluate the fidelity of a distorted video composed of some continuous images over time. Using the SSIM index, an approach to measure the statistical properties locally is more appropriate for video quality assessment. Suppose the total number of images in a video is *M*, a video data matrix is denoted by *X*

(7) $$X=\left[\begin{array}{c} X_{1}\\ X_{2}\\ \vdots \\ X_{M} \end{array}\right]=\left[\begin{array}{cccc} x_{11} & x_{12} & \cdots & x_{1N}\\ x_{21} & x_{22} & \cdots & x_{2N}\\ \vdots & \vdots & \ddots & \vdots \\ x_{M1} & x_{M2} & \cdots & x_{MN} \end{array}\right]$$

Each row of *X* corresponds to an image signal with *N* elements. With the use of SSIM index, the video fidelity index between the distorted video *X* and the reference video *Y* can be obtained as follow:

(8) $$\text{SSIM}\left(X,Y\right)=\frac{1}{M}\overset{M}{\underset{j=1}{\text{∑}}}\text{SSIM}\left(X_{j},Y_{j}\right)$$

where *X_j_* = [*x*_*j*1_,*x*_*j*2_, ⋯, *x_jN_*] and *Y_j_* = [*y*_*j*1_,*y*_*j*2_, ⋯, *y_jN_*].

## 4. The Structure Fidelity Data Collection Framework

Our SFDC framework is built on a two-layer network architecture and consists of two phases: learning and data collection. During the learning phase that aims to analyze the collected data, we need to consider how best to create clusters within the network so as to make the most of the utility of spatial correlation between nodes in the structural similarity assessment. Here, we will first introduce the clustering procedure performed for the cluster construction and the data-driven cluster head selection, which are very important basis for our data collection framework and determine the efficiency of the subsequent node scheduling scheme within cluster. Finally, we will describe the details of data collection phase.

### 4.1. Cluster Construction

Due to the prevalence of spatial correlation in environmental phenomena, the over-deployed sensor nodes used to sense environment will be highly correlated. If we group nodes with similar sense quality into the same clusters, it will be more efficient at exploiting the correlation of data. Naturally, we consider the physical distance as one of the metrics of the close relationship between any two nodes. Many mathematical models of spatial correlated sensor networks data have been established in [29–31], where the variogram (also called semivariance) that reveals the average square difference between the readings of sensor nodes is used to characterize spatial correlation in data.

Given a two dimensional stationary process *S*(*x, y*), where (*x, y*) is the geographic coordinate, the variogram is denoted as follow:

(9) $$\gamma =\frac{1}{2}E\left[{\left(S\left(x_{1},y_{1}\right)-S\left(x_{2},y_{2}\right)\right)}^{2}\right]$$

For isotropic random processes [32,33], the variogram depends only on the Euclidean distance $r=\sqrt{{\left(x_{1}-x_{2}\right)}^{2}+{\left(y_{1}-y_{2}\right)}^{2}}$ between two nodes. The experiment results in [29] show that the variogram increases quickly with the distance grows. For large distance, the process exhibits a remarkably feature of independent and identically distribution process, where the variogram do not change with distance.

Therefore, the distance is a good indicator of correlation between nodes. However, we believe that it is insufficient in real world. The reason is that the phenomenon under observation is often influenced by multiple sources such as room air conditioners, heaters, walls and so on. Even though two nodes are physically close, the correlation between them may be less obvious.

We thus consider another statistic named Pearson's coefficient [34,35] as the supplementary metric to characterize spatial correlation in data. The Pearson's coefficient indicates the degree of linear correlation between two variables *x* and *y*, giving a rang of [−1, 1], where 1 is total positive correlation, 0 is no correlation, and −1 is total negative correlation. Here each individual sensor node can be regarded as a variable to record the change of observation in the monitored area where the sensor is located. Consequently, the formula for Pearson's coefficient when applied to two sensor variables is

(10) $$\rho_{xy}=\frac{\overset{m}{\underset{i=1}{\text{∑}}}\left(x_{i}-\bar{x}\right)\left(y_{i}-\bar{y}\right)}{\sqrt{\overset{m}{\underset{i=1}{\text{∑}}}{\left(x_{i}-\bar{x}\right)}^{2}\sqrt{\overset{m}{\underset{i=1}{\text{∑}}}{\left(y_{i}-\bar{y}\right)}^{2}}}}$$

where *m* is the number of reading samples, *x̅* and *y̅* are the average of reading samples for each sensor node. The Pearson's coefficient can identify how much one variable is linearly associated with another. In other word, the areas sensed by two nodes with larger value of the coefficient have more similar linear relation in term of sensor readings. A known Pearson's distance is defined as

(11) $$d_{xy}=1-\rho_{xy}$$

From the fact that the Pearson's correlation coefficient falls between [−1, 1], it is seen that the Pearson's distance lies in [0, 2].

We address the cluster construction problem by using the Euclidean distance as the primary metric and the Pearson's distance between node readings as the secondary metric, where the correlation of sensor nodes is depicts in term of the variance and the linear relation of their readings in the past. Given a node set *G*, we need to partition *G* into *N* disjoint clusters *C* such that sensor nodes within the same cluster are correlated. The details of the cluster formation applied in the sink are described in Algorithm 1.

We define the threshold *max_dis* as a tunable parameter that roughly determines the diameter of a cluster and the number of clusters. The predefined parameter changes inversely with the number of clusters or the percent of nodes being cluster heads, which means that it will be set to a small value when the percent of cluster heads in the network is large and vice versa. In LEACH [10], authors indicate that there exists an optimal percent *α* of nodes that should be cluster heads for a suitable energy dissipation consideration. *α* = 0.05 for LEACH. In general, the predefined parameter *max_dis* is less than the communication radius of sensor node, where we only consider the senor nodes with the same communication ability for the homogeneous network. In Algorithm 1, we first put the nodes close to each other in term of spatial distance together, and then sort them in order of the Pearson's distance. Only the nodes within the communication range *R_CH_* of the cluster head join in the new cluster. The algorithm outputs a set of clusters in which all nodes are covered. The cluster head selection process is described in next section.

### 4.2. Cluster Head Selection

Prior to the cluster head selection, we need to build a data transmission model between member nodes and cluster head. Normally, all nodes including the CH in the cluster sense the environment, and then member nodes report their readings to the CH via a hop distance.

Let *X_M_*_×_*_N_* as described in Equation (7) represents the dataset received by the CH, where *M* and *N* denote the discrete time domain and the number of sensor nodes in the cluster respectively. Consider a node scheduling method, only *n* ≤ *N* sensor nodes are in a working state. In the case, the CH only receives *M*× *n* readings from the working node. In order to measure the SSIM index between the original dataset *X* and the later dataset *Y*, we need to find a way to fill the *M* × (*N* − *n*) remainder readings in *Y*. A polished model is designed for that, where all missing readings are replaced by the readings from CH without any increase in traffic. To reduce the error generated by this replacement, the mean squared deviations (MSD) on the readings between the CH and other nodes must be minimized. Based on the principle, the node with the minimal mean squared deviation (*Min_MSD*) will be selected as the CH. Due to member nodes in the cluster can receive and send the readings with each other, each node makes an independent decision based on the mean squared deviation value as to whether to be the CH.

A *Min_MSD* based distributed cluster head selection algorithm named *Min_MSD* approach is described in Algorithm 1.

**Algorithm 1** Cluster formation and cluster head selection
**Input:** a node set *G***Output:** a set of clusters covering the set *G* and the cluster head for each cluster1:Set all nodes in *G* as uncovered2:**while**
*G* is not null **do**3: Randomly pick up the node *υ* from *G*4: Pick up all the nodes distance from less than the threshold *max_dis* and put them into a list of *S*5: **for** every node *i* in the list *S*
**do**6:   
$\text{MSD}\left(i\right)=\frac{1}{M}\overset{M}{\underset{j=1}{\text{∑}}}\underset{k\in S,k\neq i}{\text{∑}}{\left(x_{ji}-x_{jk}\right)}^{2}$7: **end for**8: Cluster head *CH* = {*i*∣ min(*MSD*(*i*))}9: Calculate the Pearson's distance of each node in *S* and *CH*10: Sort the nodes in *S* according to the increasing order of the Pearson's distance value11: Create a new cluster *C* including only *CH*12: **while**
*S* is not null **do**13:   Pick up the next node *s* from *S* in turn14:  **if**
*s* is within the communication radius *R_CH_* of the *CH*
**then**15:   Put *s* into the cluster *C*16:  **end if**17: **end while**18: Output the cluster *C* and cluster head *CH*19: Remove all nodes in *C* from *G*20:**end while**


Algorithm 1 works by combining two main operations: cluster formation and cluster head selection. Two metrics (Euclidean and Pearson's distance) are used to group together nodes with high spatial correlation to form a cluster, and cluster head selection aims to minimize the structure distortion generated by our data transmission model between member nodes and cluster head. First, the geographically close nodes are roughly divided into a list of nodes *S*, where the node with the minimal mean squared deviation is elected as the cluster head. Second, the nodes in list *S* are sorted according to the increasing order of the Pearson's distance between them and the elected cluster head. Finally, the nodes both with low Pearson's distance value and within the communication radius of the cluster head have a better chance of joining the new cluster. It is important to emphasize that a greedy strategy is adopted in Algorithm 1 to ensure that all nodes are covered.

### 4.3. Nodes Scheduling Scheme Based on the Structural Similarity Index

After the cluster construction, the member nodes send all readings to the single cluster head node. In this case, the cluster head creates continuous images over time, where each image is composed of the readings sensed by all nodes at an instant. For a nodes scheduling scheme, only a subset of nodes is selected to work and sample, while the rest of sensor nodes go to sleep to save energy. Based on our data transmission model, the readings of unselected sensor nodes are replaced by the cluster head readings from the same time. The replaced dataset denoted *Y_M_*_×_*_N_* can provide a good structural similarity index over the monitored area during the subsequent data collection phase.

The main goal of our scheduling scheme is to enable the nodes with greater contribution to the SSIM index to enter active mode. Since the SSIM index is a way to measure the image structure quality from three different aspects: mean distortion, variance distortion and loss correlation, it is hard to calculate each individual node's contribution to overall structure of collected data. From the property (3) of the SSIM index, however, we know that the maximal value 1 is achieved only when the image signals being compared are identical. In other words, the closer they are to each other, the closer SSIM is to 1. The increase in the deviation between the distorted image and the reference image inevitably leads to a decrease in the SSIM index. Based on the replacement principle as described above, our nodes scheduling scheme tries to sort the member nodes in decreasing order of the deviation between the readings of them and the cluster head, and add the sensor node one by one into the active node set until the predefined structure fidelity is met, such that the SSIM index will undergo the largest increase as the addition of the candidate node.

We present a heuristic algorithm to tackle the problem of active nodes selection. Initially, the active node subset *A* only includes the cluster head node while all elements in the member node set *Q* are unselected. At this point, the cluster head generates a data sequence viewed as the first image with the largest distorition $Y_{i}^{1}=\left[x_{iC},x_{iC},\cdots ,x_{iC}\right]$, where *x_iC_* denote the reading from the cluster head at the time *i* and all readings of member nodes are replaced by *x_iC_*. Let *x_ij_* be the reading of the *j*-th member node at the time *i*, and the reference image is represented by *X_i_* = [*x_i_*_1_, *x_i_*_2_, ⋯, *x_ij_*, ⋯, *x*_i(*N*−1)_, *x_iC_*]. The node *j* among the set *Q* is selected into the active set *A* if and only if *x_ij_* is with the biggest difference with *x_iC_*. Then, the node *j* is removed from the set *Q* and the second distorted image is given by $Y_{i}^{2}=\left[x_{iC},x_{iC},\cdots ,x_{ij},\cdots ,x_{iC},x_{iC}\right]$. The value of $\text{SSIM}\left(X_{i},Y_{i}^{2}\right)$ can be calculated through Equation (6). So repeatedly until a minimal subset is built while meeting the predefined threshold. $Y_{i}^{N}$ is equal to *X_i_*, where all nodes will be incorporated into the active node set *A* when the threshold is set to 1.

Here we define the contribution rate of node to the structural similarity.

#### Definition 1

*Let* $Y_{i}^{k}$ *be the nodes data sequence after inserting the reading x_ij_ of the j-th node at the time i into* $Y_{i}^{k-1}$, *the contribution rate of node j to the structure of reference node data sequence X_i_ is defined as:*

(12) $$\alpha_{j}=\text{SSIM}\left(X_{i},Y_{i}^{k}\right)-\text{SSIM}\left(X_{i},Y_{i}^{k-1}\right)$$

*where*

$$\text{SSIM}\left(X_{i},Y_{i}^{k}\right)=\frac{4\mu_{X_{i}}\mu_{Y_{i}^{k}}\sigma_{X_{i}Y_{i}^{k}}}{\left(\mu_{X_{i}}^{2}+\mu_{Y_{i}^{k}}^{2}\right)\left(\sigma_{X_{i}}^{2}+\sigma_{Y_{i}^{k}}^{2}\right)}$$

*and*

$$\text{SSIM}\left(X_{i},Y_{i}^{k-1}\right)=\frac{4\mu_{X_{i}}\mu_{Y_{i}^{k-1}}\sigma_{X_{i}Y_{i}^{k-1}}}{\left(\mu_{X_{i}}^{2}+\mu_{Y_{i}^{k-1}}^{2}\right)\left(\sigma_{X_{i}}^{2}+\sigma_{Y_{i}^{k-1}}^{2}\right)}$$

In order to statistically assess the node contribution rate, the deviation and SSIM index can be extended over multiple time instances. We still use *X* to denote the data sequences of all nodes as described in Equation (13). The cluster head generates the first data matrix *Y*^1^ represented by Equation (14) where the elements for each row are identical and equal to *x_iC_* (1 ≤ *i* ≤ *M*).

(13) $$X=\left[\begin{array}{c} X_{1}\\ X_{2}\\ \vdots \\ X_{M} \end{array}\right]=\left[\begin{array}{ccccc} x_{11} & x_{12} & \cdots & x_{1(N-1)} & x_{1C}\\ x_{21} & x_{22} & \cdots & x_{2(N-1)} & x_{2C}\\ \vdots & \vdots & \ddots & \vdots & \vdots \\ x_{M1} & x_{M2} & \ldots & x_{M(N-1)} & x_{MC} \end{array}\right]$$

(14) $$Y^{1}=\left[\begin{array}{c} Y_{1}^{1}\\ Y_{2}^{1}\\ \vdots \\ Y_{M}^{1} \end{array}\right]={\left[\begin{array}{ccccc} x_{1C} & x_{1C} & \cdots & x_{1C} & x_{1C}\\ x_{2C} & x_{2C} & \cdots & x_{2C} & x_{2C}\\ \vdots & \vdots & \ddots & \vdots & \vdots \\ x_{MC} & x_{MC} & \ldots & x_{MC} & x_{MC} \end{array}\right]}_{M\times N}$$

If the MSD of a candidate node and the cluster head node is maximal among all such nodes, then the descriptive power of the active nodes set will maximally grow if the candidate is added into it. The values of MSD for all member nodes and the cluster head are known to the cluster head based on the cluster head selection procedure, without any increase in the computing load. We can easily obtain the value of *SSIM* (*X, Y*) defined in Equation (8) to evaluate the fidelity of the data from active nodes set. We named the active nodes selection as the *Max_MSD* approach. The detailed algorithm for finding the set of active nodes with a minimal number of nodes in a cluster is presented in Algorithm 2:

**Algorithm 2** Active nodes selection
**Input:** predefined fidelity threshold *δ***Output:** a set of active nodes *A*1:*A*={*CH* node}, *Ā*={all member nodes}2*:**k* = 13:**while**
*SSIM* (*X, Y^k^*)< *δ*
**do**4: Candidate node *j* = {*i*∣ max(*MSD*(*i, CH*)), *i* ∈ *Ā*}5: Put *j* into *A* and remove from *Ā*6: *k* = *k* + 17:**end while**8:Output the node set *A*

### 4.4. Data Collection

Once clusters are formed and active sensors serving as samplers to collect data are selected, no-sampler nodes enter the energy-efficient sleep mode without much degradation of structure fidelity. Note that with a fixed number of sampler nodes determined throughout the data collection phase, it is possible that the fidelity is not always superior to the preset threshold. In essence, the phenomenon that WSN is sensing is in a constant state of flux and continual change. Even an anomaly may occur in a particular time slot, for example a sudden flood, forest fire or earthquake involved in the event detection problem. More likely, the intra-cluster nodes relationships may imperceptibly change due to the slight and gradual changes in environment. Such the observation on temperature and humidity is clearly affected by seasonal changes. These changes can lead to the negative effects on the performance of our data collection framework. The first is that the nodes in a cluster no longer have a close spatial correlation. Furthermore, sensor nodes have a different sort for the data deviation compared to previous data, which would further affect the quality of cluster head and sampler nodes selection.

We define the global parameter denoted by *T_d_* as the period of data collection and *T_l_* (*i.e.*, *M*) as the period of learning. The setting of *T_d_* is mainly for consideration of two aspects. On the one hand, the sampler nodes consume more energy compared to the sleeping nodes, since they carry out the task of sensing and report the readings. The cluster head node has additional responsibility for collecting the data from all sampler nodes in the cluster and forwarding them to the sink. In this case, energy dissipation is not balanced especially for the large number of sampler nodes. Moreover, the spatial correlation of nodes may change with the change of sensor readings. As a result, the nodes scheduling scheme fails to keep stability in a long run. Consequently, the value of *T_d_* should be small enough to balance the energy consumption and maintain the correlation between different nodes. On the other hand, too small value of *T_d_* can not result in a substantial saving in energy consumption. Its value is expected to be much large than the learning period *T_l_*. Thus, the data collection period should be dynamically adjusted to maintain the stability of correlation structure in the cluster in response to environmental changes.

In order to deal with the dynamics, a feasible solution to adaptively adjust the data collection period is that the cluster head node broadcast a “relearning” message to all member nodes when the drastic deterioration in sensor readings happen. In our nodes scheduling scheme, what we are most concerned is any trend changes occurred in the monitored field. Therefore, we define a metric to approximately measure the cluster trend based on the sensor time sequences.

#### Definition 2

*Two sensor nodes are isotonic if there is* $\frac{m_{1}}{m}>t$ *for their time sequences* {*x*_1_, *x*_2_ ⋯, *x_m_*} *and* {*y*_1_, *y*_2_, ⋯, *y_m_*}, *where m*_1_ *is the number of pairs* (*x_i_, y_i_*) *in the time sequences that satisfy* (*x_i_−x_i−_*_1_) (*y_i−_y_i−_*_1_) ≥ 0, 2 ≤ *i* ≤ *m*.

#### Definition 3

*The cluster trend is deteriorating if any two nodes in the cluster are not isotonic*.

According to Definition 2, the isotonicity means that the value of a senor will increase/decrease with the value of another correlated senor increase/decrease in most of the time. As a more precise indicator to highlight the impact of sensor readings' change on data structure, the isotonicity is different from the former Pearson's coefficient, which roughly classifies the sensor nodes from the perspective of linear relation and thus more suitable to be used at the time of cluster launch. The value of *t* depend on the requirement of the application at hand. Once the trend deterioration based on Definition 3 is identified, the cluster head broadcasts a “relearning” message that means terminating the data collection procedure. After receiving the message, awakened no-sampler and working sampler nodes start a new learn phase only including selection of cluster head and active nodes. If the trend deterioration occurs during the learning period, the cluster head broadcasts a increased value of *T_l_* to all member nodes to guarantee the accuracy of the video fidelity index. Moreover, if the percentage of cluster required relearning is more than a value (*i.e.*, 50%), the sink broadcasts a similar relearning message to all sensor nodes via the hierarchical routing. The complete learning process will be performed in the whole network.

Our method by adjusting the data collection period based on the cluster deterioration avoids the structure index instability resulting from the change of the inter-node relationships, thus ensuring the adaptation of the data collection system without human intervention.

### 4.5. Energy Consumption

Unlike other models using the radio distance in calculating the energy cost, we analyze the energy consumption from the perspective of the number of working/sleeping sensor nodes in unit of cluster on the learning and data collection phases, since our approach aims to energy saving by reducing the number of nodes that have no need to be active. First, all member nodes send the readings to the cluster head node during the learning phase, thus there is not any sleeping node (or energy conservation). The energy cost in a cluster with *N* nodes during the learning phase is

(15) $$E_{l}=T_{l}N_{e}$$

where *e* is the energy cost on transmitting a unit data between two nodes.

During the data collection phase, the energy cost in the cluster is

(16) $$E_{d}=T_{d}N_{a}e$$

where *N_a_* is the number of active nodes.

Compared the energy consumption without any nodes scheduling, the percentage on energy saving within a cluster during a cycle (*i.e.*, learning and data collection) is

(17) $$P_{E}=1-\frac{T_{l}Ne+N_{d}N_{a}e}{\left(T_{l}+T_{d}\right)Ne}=\frac{T_{d}\left(N-N_{a}\right)}{\left(T_{l}+T_{d}\right)N}=\frac{T_{d}}{T_{l}+T_{d}}\frac{N-N_{a}}{N}$$

Equation (17) indicates that a good energy conservation can be achieved by extending the data collection period *T_d_* and reducing the number of the working nodes *N_a_*. *T_d_* and *N_a_* depend on the sensed environment and the pre-defined fidelity threshold respectively. We will evaluate them in the following simulations.

## 5. Performance Evaluation

### 5.1. Real Dataset

**Figure 1.**
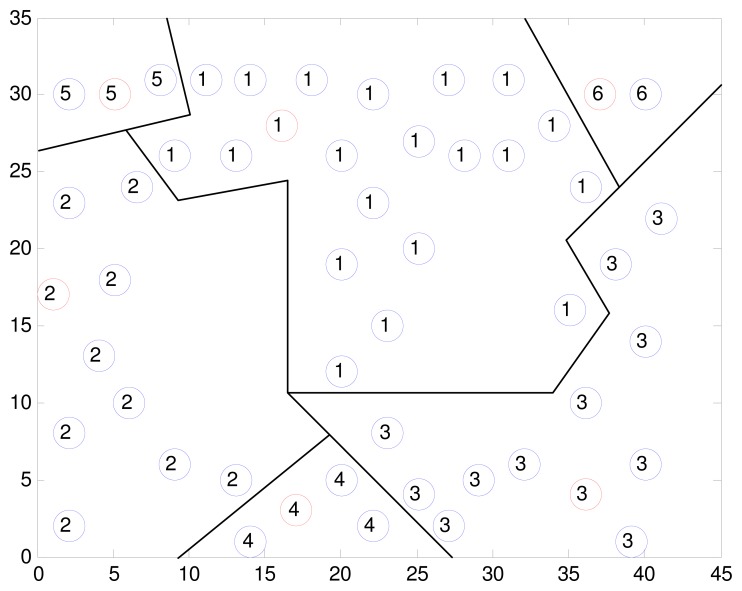
A clustering map is produced by implementing the Algorithm 1.

The experiment dataset plays an important role in the simulation and implementation of the structure fidelity data collection framework. Thus, we used the real world dataset from the Intel Berkeley Research lab [36] as the evaluation object. The data set including humidity, temperature, light, and voltage values is collected from 54 Mica2Dot sensor nodes deployed in the lab layout for a period of one-month. In the publicly available data set, we selected the temperature readings sampled every thirty one seconds for all nodes to perform a data analysis. Since 2 sensor nodes are without data available for a long period of observation in the dataset, only 52 nodes are used in our experiment. As shown in Figure 1, the location information of all nodes is fixed at configuration time and hence the distance between nodes can be calculated. The *X* and *Y* axis in Figure 1 represent coordinates of sensors in meters relative to the lower left corner of the lab.

#### 5.1.1. The Correctness of Clustering with SFDC

To group together nodes with high spatial correlation to form a cluster, the Algorithm 1 is designed to heuristically obtain a rough approximation on correlation based on two indexes of geographical distance and Pearson's distance. The network is partitioned to several subareas in which the distance of any two nodes is smaller than *max_dis*. As the growth in sensor network scale, *max_dis* will be set to be a larger value such that an optimal number of clusters is achieved. For a fixed scale sensor network, the smaller *max_dis* leads to the more number of clusters. The optimal number of clusters by minimizing the communication cost of the network presented in [10] can be used to be a reference.

Another parameter needed to be supplied is the communication radius *R_CH_* of cluster head nodes and it depends on the application as well as the sensing hardware of sensor node. In general, the cluster heads have a larger communication radius than member nodes since they receive all data from member nodes and perform long range transmissions to the remote base station. We assume that all cluster head nodes have the same value of *R_CH_* to guarantee the expedite communication within their clusters.

As an example, Figure 1 illustrates the output of one of our clustering experiments with the default parameters *max_dis* = 15, *R_CH_* = 25, *M* = 50, *N* = 52, where the sensor network is organized into 6 clusters. All nodes marked with the same sequence number belong to the same cluster, and the cluster head nodes are marked in red. It can be see that the nodes adjacent to each other are grouped into one cluster and member nodes are close to the cluster heads in term of the Pearson's distance. Due to the Pearson's distance based on the historical readings is time-varying, the clustering map is not constant at different data collection phase. And hence it is reasonable to make the geographical distance that is constant as the primary metric without compromising the Pearson's distance.

#### 5.1.2. The Fidelity without the Dynamical Adjustment of *T_d_*

In this section, we will evaluate the fidelity without the dynamical adjustment of *T_d_* during data collection by following the clustering map in Figure 1, and try to get some qualitative relations between the fidelity, the number of nodes and the sleeping node ratio. By default, the predefined fidelity threshold *δ* is set to 90%.

**Figure 2.**
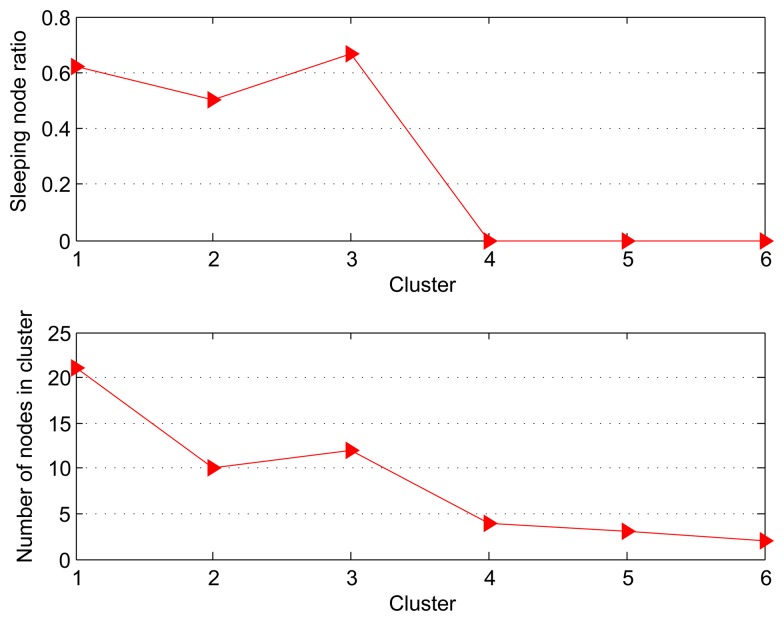
SNR *vs.* Nodes number.

Figure 2 shows that the sleeping node ratio (SNR) and nodes number for each cluster. SNR is defined by the following equation:

(18) $$\text{SNR}=1-\frac{\text{Number of active nodes}}{\text{Total number of nodes}},$$

which describes the ratio of the sleeping node for each cluster. In other words, the larger SNR, the more idle nodes that means the more energy conservation. Intuitively, when the number of nodes in the cluster decreases significantly, the SNR also appears to noteworthy decline. As we see in Figure 2, the SNR is more than 60% for the nodes in Cluster 1. We also note that the worst SNR 0 is obtained by Cluster 6 with the minimum number of nodes. Therefore, our SFDC framework is more suitable for the large scale dense WSNs in order to achieve a better effect on energy saving. The conclusion is experimentally verified by running the Algorithm 1 multiple times.

**Figure 3.**
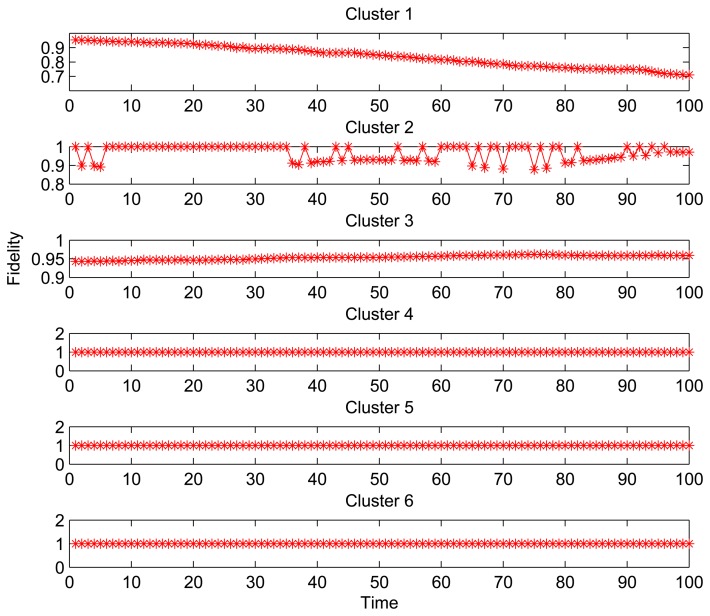
Fidelity curves without the dynamical adjustment of data collection period.

Figure 3 shows the fidelity curves for 6 clusters with different size for 100 time slots (thirty one seconds per time slot). For the Clusters 1 and 2 with more number of nodes, the fidelity curves show a decline and instability. On the other hand, for Clusters 3–6 with fewer nodes, the fidelities are superior to the threshold throughout the data collection phase. Especially for Clusters 4–6 in which all nodes are active, it can be found that the fidelities during data collection are always 100%. It happens because the structure fidelity is less or not susceptible to the reading changes from the observed area where the less sleeping nodes are located. Obviously, the increase on the number of sleeping nodes leads to the performance degradation (structure distortion), which is amplified when the number and density of node deployment are increased.

Therefore, we notice that there is a trade-off between the fidelity in the data structure and the SNR. This raises the necessity to perform the dynamical adjustment of *T_d_* to achieve large SNR while guaranteeing the low distortion.

#### 5.1.3. The Correctness of Cluster Head and Active Nodes Selection

The preceding discussion describes that our data collection framework benefits the large scale dense WSNs in term of SNR. To exploiting the potential of SFDC, we use all 52 sensor nodes as a single cluster to perform the following simulations.

**Figure 4.**
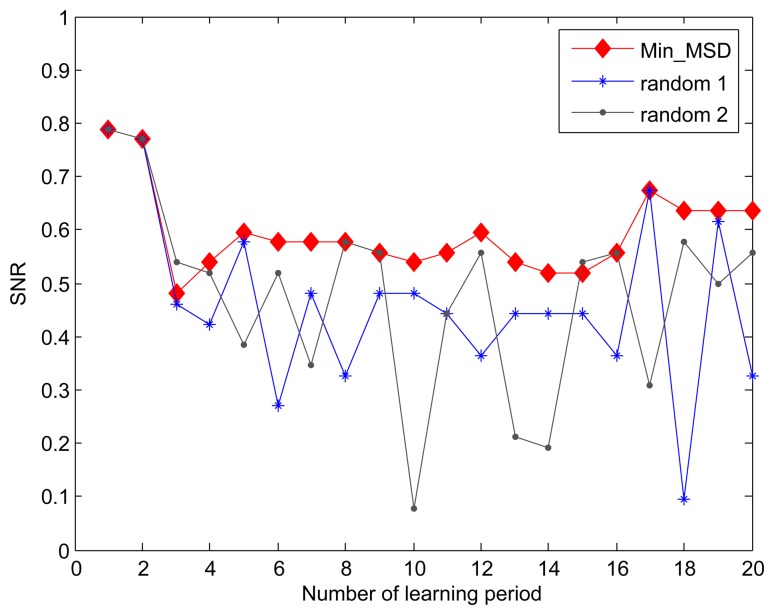
Effect of cluster head selection using the *Min_MSD* and *random* approaches on SNR.

For the purpose of comparison, we introduce a variant of our *Min_MSD* approach-*random* approach, where the cluster head node is randomly selected in the cluster. When the fidelity threshold is set to 90%, we compare the SNR for *Min_MSD* and *random* approaches through 20 learning periods. As shown in Figure 4, the *Min_MSD* presents better SNR than using the *random* approach throughout all learning periods. Based on the design in Algorithm 1, the result can be easily explained by the fact that the smaller deviation produces the smaller distortion in our data transmission model.

**Figure 5.**
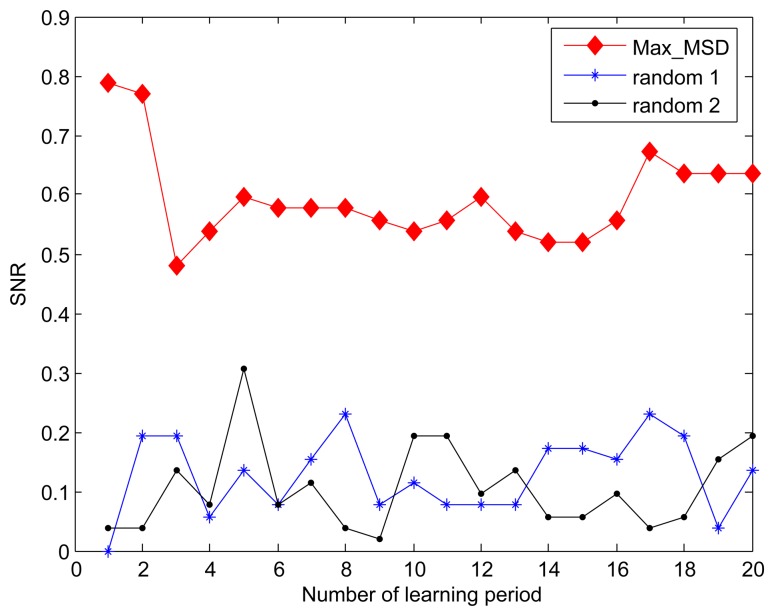
Effect of active nodes selection using the *Max_MSD* and *random* approaches on SNR.

In previous evaluation, both of *Min_MSD* and random approaches used the same active nodes selection scheme as described in Algorithm 2 to derive the SNR. For simplicity, we also name the random active node selection the *random* approach. Figure 5 shows the effect of active nodes selection using the *Max_MSD* and *random* approaches on SNR. It is obvious that the greater SNR is achieved by our *Max_MSD* approach since each selected active node can eliminate the most difference between the current and original active nodes set. Another result viewed from the Figures 4 and 5 is that the performance degradation of *random* approach shows a distinct advantage provided by *Max_MSD* in terms of SNR, compared to that of the *Min_MSD*. The reason is that more active nodes with maximal MSD is selected instead of completely randomized nodes.

#### 5.1.4. Node Contribution Rate

**Figure 6.**
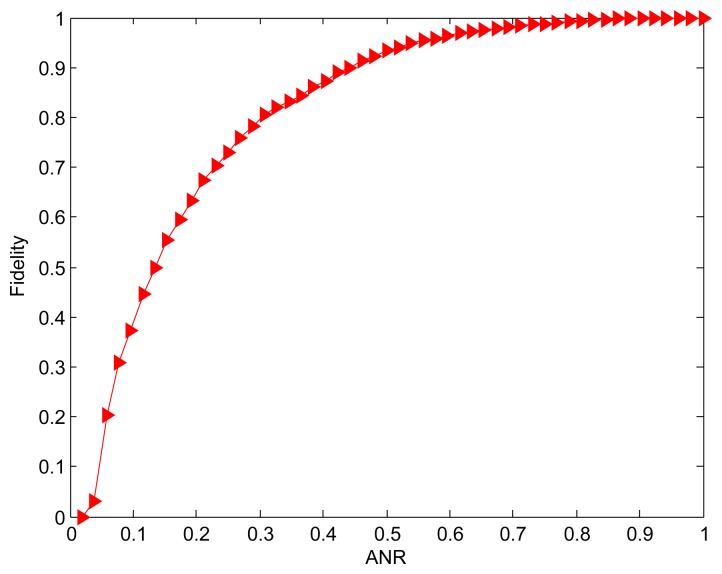
Structure fidelity *vs.* ANR.

In this evaluation, we try to examine the node contribution rate *α* to structure fidelity in order to provide a way to determine the fidelity threshold in practice. Figure 6 shows the structure fidelity when all 52 nodes are added into the active nodes set one by one. It can be observed that the fidelity monotonically increases with the active node ratio (ANR), which is the ratio of active nodes to all nodes. Consequently, the fidelity is optimal (100%) when the ANR is equal to 1. This is no surprise because more selected active nodes means smaller distortion. Based on Definition 1, we calculate the contribution rate for different number of nodes. The result is given in Table 1. The initial 22 active nodes with 42.31% ANR contribute 90.56% of fidelity, while the subsequent three active node sets only provide 6.42%, 2.46%, 0.56% of the whole fidelity, respectively. For the evaluated scenarios, therefore, 61% of nodes need to work when the target fidelity is set to a high value 96%. That means it is possible to save 39% energy on nodes dissipation while only losing 4% fidelity.

Based on above analysis, we can draw the conclusion that the spatial redundancy in the experimental scenario is high since last 30 nodes representing about 58% of all nodes account for less than 10% of fidelity. Thus, a way is introduced for evaluating the level of nodes redundancy. For the WSNs with high spatial redundancy, the satisfying energy saving can be achieved while the expected threshold is met.

#### 5.1.5. Effect of Adaptive Data Collection

The principal remaining problem is that the underlying physical phenomenon is complex and not constant. The only information available is what can be learned from all reported readings. Then the adaptive data collection will be executed in response to the spatial correlation changes. Once the cluster head detects that a cluster should be relearned, it asks all sensor nodes in the corresponding cluster to collect the reading simultaneously. In the worst case, the sink can re-cluster the whole network when most clusters trend in the network is deteriorating.

**Figure 7.**
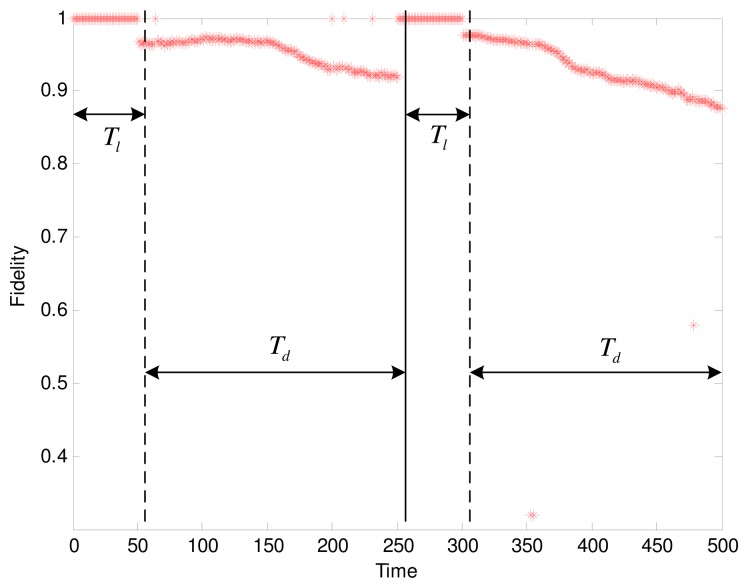
The changing fidelity during the data collection.

We experimentally test our adaptive data collection scheme to observe the changing fidelity. The settings of parameters are as follows: *t* = 0.4, *T_l_* = 50, *T_d_* = 4*T_l_* = 200. Based on the results in the previous section, the SNR is 39% when the fidelity threshold is set to 96%. By evaluating the isotonicity between nodes to judge the cluster trend, we get the result shown in Figure 7. The fidelity during the learning phase keeps a constant value of 1. In two periods of data collection, the fidelity curves show a clear decline. However, after each round of data collection, the cluster starts a relearning process when the cluster head detected the deterioration of cluster trend. Consequently, the only suitable candidate is elected as the new cluster head by executing the Algorithm 1. Afterwards, an updated working schedule is created by the cluster head, thus avoiding the continued decline in the fidelity. As we can see in Figure 7, the result is verified that the structure fidelity with the adaptive data collection is acceptable.

According the Equation (17), 31.2% energy for the network with 52 sensor nodes is saved. We can set the more strict value of *t* or shorten the period of data collection to enhance the fidelity, however, that will not achieve the significant reductions in energy. The tradeoff between the fidelity and the energy saving depend on the tolerance of distortion in the particular sensor applications. Notice that there are several outliers in the second *T_d_*, where much lower fidelity scores than they should supply are given. In fact, most of these significant outliers correspond to the sensor readings with large global motions at some instants. An outlier detection approach is beyond the scope of this paper, which is primarily aimed at the structural distortion.

### 5.2. Synthetic Data

To verify our data collection framework for the large scale WSNs, we adopted the environmental model in [37] for synthetic data generation that provides a possibility for the realism of simulated data. Two sets of sensor nodes each with the number of 500 and 1000 are scattered randomly in a square field, in which an unknown number of diffusion heat sources move randomly at a set of positions and data was collected for every time instant at each sensor position. Modeling of the temperature variations due to heat conduction can be obtained by using the following partial differential equation:

(21) $$\frac{\partial V\left(X,t\right)}{\partial \left(t\right)}-k\Delta V\left(X,t\right)=Q,$$

where *V*(*X, t*) is the temperature value at the coordinate point *X*(*x*_1_, *x_2_*) and time *t, k* is the diffusion coefficient for heat propagation, $\Delta V\left(X,t\right)={\text{∑}}_{i=1}^{2}\frac{\partial^{2}V\left(X,t\right)}{\partial^{2}x_{i}}$ and *Q* is the heat source in Joule.

**Figure 8.**
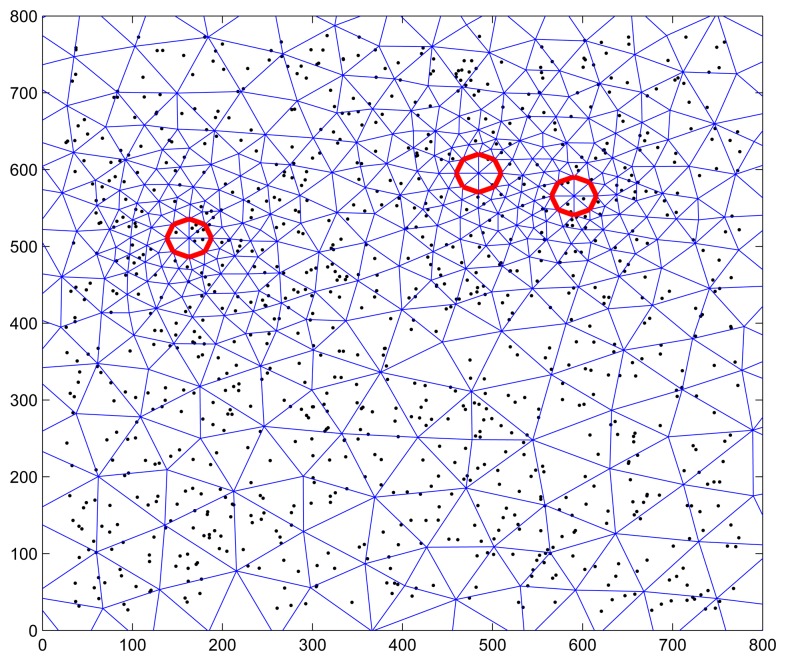
Random scenario with 3 heat sources and 1000 sensor nodes.

By setting the following parameters in Matlab simulation: *k* = 0.1 *m*^2^/*instant*, 3 heat sources of *Q* = 200 *W* with 20 random positions and 20 *m* radius, 2000 observations for every position (or sensor node) were collected. Figure 8 shows the scenario of 1000 sensor nodes with triangular meshes, in which the red circle indicates the position of head resource at a time. Since the correctness that cluster head and active nodes selected in the learning phase of SFDC is validated in Section 5.1.3, more attention is paid to the performance of ANR and adaptive data collection in the case when a large number of sensor nodes are used in the simulation.

### 5.2.1. Node Contribution and Active Node Ratio (ANR)

**Figure 9.**
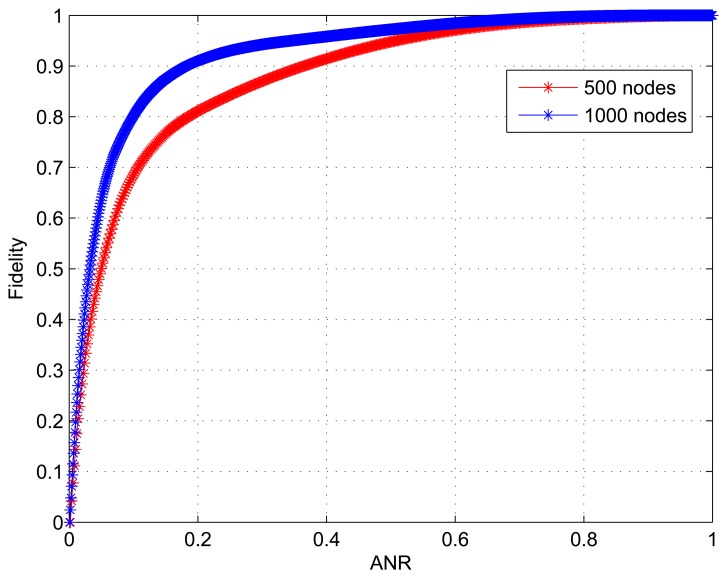
Structure fidelity vs. ANR.

We conduct simulations for two scenarios: Scenario 1 with 500 nodes and Scenario 2 with 1000 nodes deployed over an area of size 800 × 800 *m*^2^. Figure 9 shows how the structure fidelity change over ANR and the corresponding statistic is given in Table 2. As we can see, the results that the fidelity monotonically increases with ANR are similar to those in Figure 6. However, it is obvious that there are differences between the two curves in Figure 9. For Scenario 1 the initial 183 active nodes accounting for 36.6% of 500 nodes contribute 90.03% of the whole fidelity, while for Scenario 2 the initial 174 active nodes only accounting for 17.4% of 1000 nodes also provide 90.01% of the whole fidelity. On the other hand, we can see from the last column in Table 2 that 35% nodes for Scenario 2 and 18% nodes for Scenario 1 do not really need to work when 1% distortion is permitted. Because of the higher node redundancy, we conclude that the large scale dense WSNs have an advantage in the sleeping node ratio (SNR) by using our data collection approach.

### 5.2.2. Effect of Adaptive Data Collection

**Figure 10.**
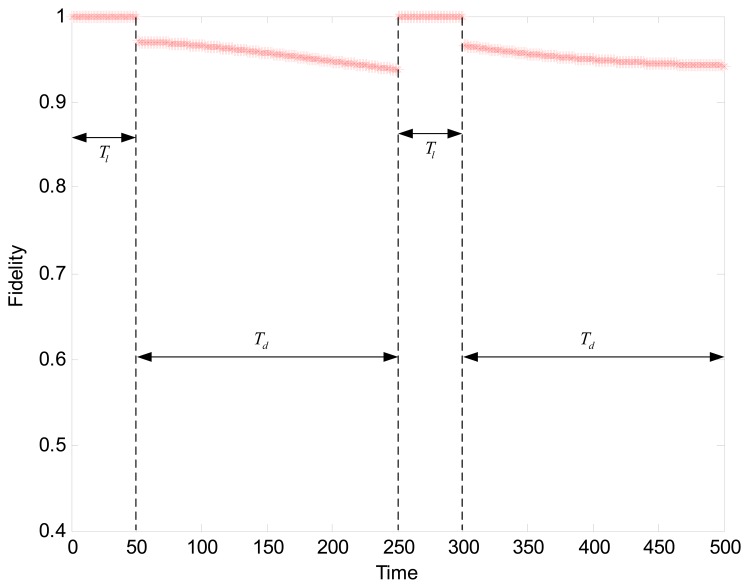
The changing fidelity for the scenario with 1000 nodes during the data collection.

Figure 10 shows the changing fidelity for Scenario 2 during the data collection by using the same parameter settings in Figure 7, *i.e.*, *t* = 0.4, *T_l_* = 50, *T_d_* = 4*T_l_* = 200. During the learning time of *T_l_*, about 35.8% nodes can be determined to be active when the fidelity threshold is set to 96%. Then a fidelity decline from 1 to 96% can be found in Figure 10 because only 35.8% member nodes communicate with the cluster head. With the change in the spatial correlation of nodes over time, there is a slight and continuing decrease in the fidelity. When the cluster head detected that the cluster trend is deteriorating, the cluster head and active nodes will be reselected to still maintain a high level of fidelity. Compared to Figure 7, in addition, there is not any fidelity outlier appearing in Figure 10 because of the ideal property of synthetic data.

We also calculated the energy saving for both of two scenario by the Equation (17) and got the following results: *P_E_* = 32.8% for Scenario 1 and *P_E_* = 51.36% for Scenario 2. The main reason for more energy saving in Scenario 2 with a denser node deployment is that a larger SNR is achieved.

## 6. Conclusions and Future Work

Our cluster-based SFDC framework takes advantage of the structural similarity index to perform the energy-aware wake-up/sleep nodes scheduling scheme by which the spatial correlation is leveraged to save even more energy. Unlike other typical clustering techniques considering the spatial correlation, both of the Euclidean distance and the Pearson's distance are used to be two metrics of the correlation of sensor nodes in our clustering algorithm. With the node scheduling scheme, only a part of nodes in the cluster are required to work for sampling, while the rest of nodes switch to the sleep mode for energy saving. Since the active node selections are based on the features of sampling data changing over time, we dynamically adjust the data collection period against the cluster deterioration to avoid the structure index instability resulted from the change of the inter-node relationships. Simulation results show that SFDC is more suitable for the large scale dense WSNs in order to achieve a better effect on energy saving. Our clustering and node selection algorithms have remarkable advantages in the sleeping node ratio when compared to the random cluster heads and active nodes selection. We also discuss the node contribution rate and provide a way to determine the fidelity threshold in practice. Finally, the adaptive data collection scheme is proved to be an effective technique in response to the spatial correlation changes.

As future work we intend to explore the temporal correlation to further reduce energy consumption without the decline of structural fidelity. And the data prediction and compression techniques can be exploited and used to achieve the objective. In addition, we will consider the problems of sensor failure and calibration into our data collection framework in future work.

## Figures and Tables

**Table 1.** Nodes contribution rate with different active node rate.

Number of Nodes	1–22	23–32	33–42	43–52
Nodes contribution rate	0.9056	0.0642	0.0246	0.0056
ANR	0.4231	0.1923	0.1923	0.1923

**Table 2.** Nodes contribution rate with different active node rate.

500 nodes	Number of active nodes	1–183	184–300	301–410	411–500
	Nodes contribution rate	90.03%	7.09%	2.35%	0.53%
	ANR	36.6%	23.4%	22%	18%

1000 nodes	Number of active nodes	1–174	175–358	359–650	651–1000
	Nodes contribution rate	90.01%	6.05%	3.02%	0.92%
	ANR	17.4%	18.4%	29.2%	35%
